# Low Pre-Existing Gray Matter Volume in the Medial Temporal Lobe and White Matter Lesions Are Associated with Postoperative Cognitive Dysfunction after Cardiac Surgery

**DOI:** 10.1371/journal.pone.0087375

**Published:** 2014-01-27

**Authors:** Kengo Maekawa, Tomoko Baba, Sumi Otomo, Shoji Morishita, Nobushige Tamura

**Affiliations:** 1 Department of Anesthesiology, Graduate School of Medical Sciences, Kumamoto University, Kumamoto, Japan; 2 Department of Anesthesiology, Kumamoto Chuo Hospital, Kumamoto, Japan; 3 Department of Radiology, Kumamoto Chuo Hospital, Kumamoto, Japan; 4 Department of Cardiovascular Surgery, Kumamoto Chuo Hospital, Kumamoto, Japan; Massachusetts General Hospital, United States of America

## Abstract

**Objectives:**

Postoperative cognitive dysfunction (POCD) is recognized as a complication in the elderly after cardiac surgery. Imaging of the brain provides evidence of neurodegeneration in elderly patients; however, abnormalities in brain structure and their relation to POCD are uncertain. This pilot study investigated whether loss of gray matter in the bilateral medial temporal lobe (MTL), seen in preoperative MRI, was associated with POCD.

**Methods:**

Data were collected prospectively on 28 elderly patients scheduled for elective cardiac surgery. MRI of the brains of all patients were assessed for prior cerebral infarctions, and carotid and intracranial arterial stenosis. Patients also completed six neuropsychological tests of memory, attention and executive function before and after surgery. POCD was defined as an individual decrease in more than two tests of at least 1 standard deviation from the group baseline mean for that test. The degree of gray matter loss in the MTL of each patient was calculated using voxel-based morphometry with three-dimensional, T1-weighted MRI. This represented the degree of gray matter change as a Z score.

**Results:**

Postoperative cognitive dysfunction was identified in 8 of the 28 patients (29%). Patients with POCD had significantly more white matter lesions on MRI, and greater loss of gray matter in the bilateral MTL (average Z score 2.0±0.9) than patients without POCD. An analysis by stepwise logistic regression identified gray matter loss in the MTL and cerebral infarctions on MRI as independent predictors of POCD.

**Conclusions:**

These preliminary findings suggested that reduced gray matter in the bilateral MTL and white matter lesions existed in brains of elderly cardiac surgery patients who experienced POCD. Additional studies with larger sample sizes are needed to confirm these findings.

## Introduction

Postoperative cognitive dysfunction (POCD) occurs frequently (20% to 30%) in patients after cardiac surgery [1]. While cognitive function tends to improve over months to years postoperatively in affected individuals, a small proportion seem to suffer permanent injury. Pathophysiologic studies of cognitive change after cardiac surgery focused on the role of cardiopulmonary bypass (CPB), intraoperative microemboli, hypoperfusion and inflammatory response as possible causes of POCD. Though long-term, follow-up study failed to show a difference in the frequency of between patients who underwent coronary artery grafting with and without CPB [2]. Thus, the focus of research is shifting from CPB to risk factors related to the patient [3].

As is commonly known, aging is associated with structural changes in the cerebrum, including impaired cognition. It has recently been suggested that preoperative cognitive impairment may identify additional patient risk for POCD [4]–[8]. This is an important finding because the population considered most at risk for POCD is subject to various other cognitive risks, including mild cognitive impairment (MCI), Alzheimer's disease (AD), vascular dementias, and the nonspecific impact of aging. More recently, Kline et al. [9] found that after surgery elderly patients experienced an increased rate of brain atrophy during the initial evaluation interval, a time associated with enhanced risk for POCD [9]. This result suggested that brain atrophy in POCD generate new mechanistic insights.

Numerous structural magnetic resonance imaging (MRI) studies have demonstrated that atrophy of the medial temporal lobe (MTL), including the hippocampus and entorhinal cortex, is a sensitive marker of early AD [10], [11]. The hippocampus and entorhinal cortex suffer the earliest neuropathological changes in AD, and the ensuing MTL neurodegeration may be linked more directly to cognitive and clinical decline than other features of the pathological process [12].

Recently, an automated method of measuring brain atrophy has been developed. This method of voxel-based morphometry (VBM), which objectively assesses whole brain structure with voxel-by-voxel comparisons, has been developed to analyze tissue concentrations between subject groups to distinguish degenerative diseases with dementia [13], [14]. Subsequently, Hirata at al. [15] used VBM to develop an automated software program, voxel-based specific regional analysis system developed to study AD (VSRAD). Z scores of VSRAD became an indicator reflecting gray matter loss in the MTL to diagnosis AD early. They found a high accuracy (87.8%) in discriminating patients with very early AD at the MCI stage from normal control subjects [15].

The purpose of this pilot study was to investigate any relationship between gray matter loss in the MTL and cognitive decline related to cardiac surgery. We used VBM analysis to estimate gray matter loss in the MTL of consecutive patients scheduled for cardiac surgery with neuropsychological testing both before and after the surgery.

## Materials and Methods

### Patients

The Medical Ethics Committee of the Kumamoto Chuo Hospital approved the study protocol and all participants gave informed written consent, according to the Declaration of Helsinki. We prospectively enrolled a consecutive series of patients age 60 y or older who underwent elective cardiac surgery by a single surgeon from May 2010 to March 2011 that included coronary artery grafting with CPB, mitral valve repair or replacement, or aortic valve replacement. Subjects were excluded if they had a history of stroke or transient ischemic attack, if they were unable to participate in neurocognitive assessments or MRI, or if they showed a preoperative Mini-Mental State Examination (MMSE) score of less than 24 out of 30.

### MRI Scans

An MRI scan and magnetic resonance angiograph (MRA) were obtained on each patient 1 to 14 days before surgery. The MRI examinations were performed using a 1.5-Tesla system (Gyroscan Intera Achieva Nova Dual; Philips Medical Systems, The Netherlands). We evaluated existing silent brain infarcts or vascular occlusion/severe stenosis at major intracranial arteries using T2-weighted turbo spin echo sequences, turbo fluid attenuated inversion recovery and three-dimensional (3D) time-of-flight MRA. The MRI findings (fluid-attenuated inversion recovery and T2) were classified as: no infarct; single infarct with a diameter ≥3 mm; multiple infarcts at the same location (cortical, subcortical, or cerebellar); or multiple infarcts in multiple locations. The degree of stenosis of intracranial arteries was graded bilaterally from MRA as: almost normal; moderate narrowing >50%; or occluded. The degree of stenosis in the carotid arteries was graded based on MRA as: normal or mild narrowing <50%; moderate narrowing of 50–75%; severe narrowing (>75%) or obstructed [16]. A Fazekas rating scale was used to grade the lesion load of MRI hyperintensities in the white matter of the brain [17]. White matter lesions were defined as punctuate foci of MRI hyperintensities, beginning confluence of foci, and large confluent areas.

### Voxel-based MRI analysis

The degree of gray matter loss in bilateral MTL was studied using VSRAD, as developed by Hirata, et al [15]. VSRAD is a type of VBM to analyze brain volume obtained from voxel units. With this method, MRI images acquired from the subjects were formatted to gapless, 2 mm-thick transaxial images, followed by extraction of the gray matter images using SPM2 (Statistical Parametric Mapping, 2002 Edition). Anatomical standardization was used to fit each individual's brain to sample brain in 3D space to correct for differences in brain size and sharpness and facilitate intersubject averaging. The segmented gray matter images were then subjected to affine and nonlinear standardization using a template of prior gray matter. The anatomically standardized gray matter images were smoothed with an isotrophic Gaussian kernel having a total width of 12 mm at half maximum. The gray matter image of patients was compared with the mean and standard deviation (SD) of gray matter images of healthy volunteers using voxel-by-voxel Z score analysis; Z score  =  (control mean voxel score- subject voxel score)/control SD. Z scores reflected the degree of gray matter loss.

**Figure 1:**
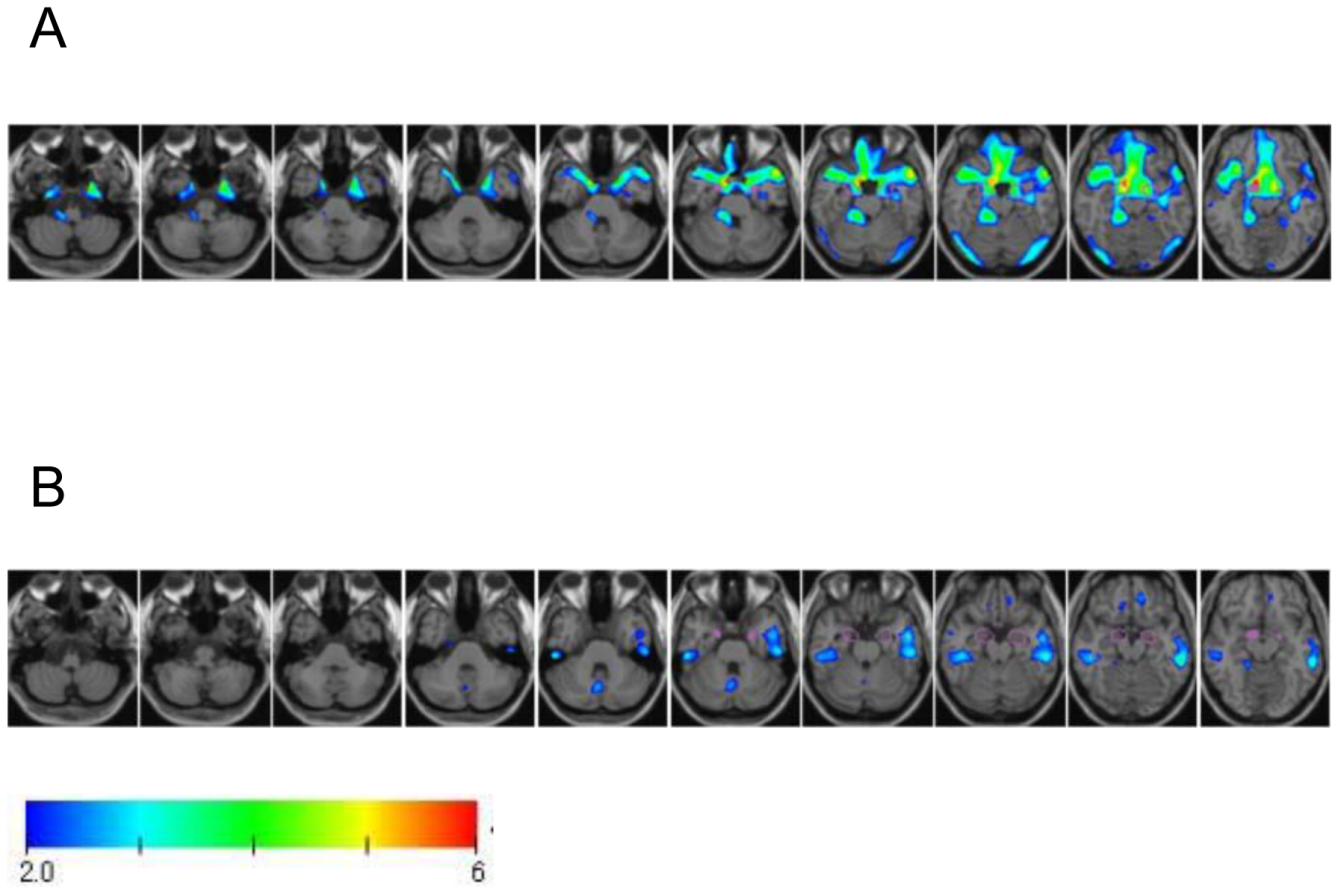
Voxel-based based specific regional analysis for Alzheimer's disease (VSRAD) analysis in a 78-year-old woman before aortic valve replacement (A) and a 68-year-old man before mitral valve repair (B). VSRAD provides a color-scaled Z score map ranging from 2.0 to 6.0 with overlaid orthogonal sections of an anatomically standardized brain template. (A) Axial VSRAD and its enlarged image at 20 mm. Gray matter was lost in the medial temporal lobe. The Z score was 3.1. (B) In contrast, there was no gray matter change in the medial temporal lobe preoperatively. Z score was 0.3.

Severity of gray matter loss in the bilateral MTL was interpreted according to the method of Hirata et al. [15] indicated in the following: Z score 0–1, hardly any gray matter loss in the bilateral MTL; 1–2, some gray matter loss; 2–3, considerable gray matter loss; 3–4, prominent gray matter loss. The results are displayed as a colored scale map superimposed on the brain (Figure 1). The database for healthy individuals includes 40 Japanese males and 40 females between 54 and 86 years of age (70.2±7.3 y.o.). The entire procedure was completed on the VSRAD program. In the present study, imaging of the entire brain volume with 3D gradient refocused echo sequence was performed for VBM analysis using the following parameters: field of view 240 mm, matrix 256×205, slice thickness 1 mm, 165 slices, repetition time [TR] 9.4 ms, echo time [TE] 4.6 ms, flip angle 10 degrees.

### Neurologic Evaluation

Cognitive testing was performed 24 h before surgery and repeated 2 weeks after surgery. The examination included several cognitive domains: (1) Orientation and global cognition were screened with the MMSE. (2) Verbal memory measured through a digit span subtest of the Wechsler Memory Scale-Revised; a task that requires short-term memory (digit span forward) and working memory (digit span backward) for verbal material. (3) Attentional performance was assessed with the digit symbol substitution test of the Wechsler Adult Intelligence Scale, in which participants transcribed number-symbol pairs under timed conditions. (4) The Kana pick-out test measured executive function. Patients read a children's fable and picked out all vowels, such as a, i, u, e and o, while also remembering the meaning of the sentences. (5) Trail-Making Test A and B, in which participants connect numbered and then alternately numbered and lettered dots in order under timed conditions to assess attention and mental flexibility. These tests were performed by one of three trained investigators who were assigned randomly to the patients. All tests were repeated postoperatively by a single investigator, who was blinded to the surgical procedure performed.

Decreased cognitive function for each test given after surgery was defined as a decrease of an individual patient's score of at least 1 SD from the baseline mean of all patients [18], [19]. This correction was calculated as the change in population score at each test point from baseline (systemic population change) with this value subtracted from each patient's change score [20]. We chose to use this 1 SD rule to define cognitive impairment in each test because this rule has been shown to be associated with fewer false positives [21], [22]. Postoperative cognitive dysfunction was defined as two or more abnormal test results [23].

### Delirium Assessments

One day after extubation all patients were screened for postoperative delirium using the Delirium Rating Scale (DRS) [24]. The DRS rates symptoms with anchored severity over a broad range. The questionnaire consists of 10 items: temporal onset (score 0–3); perceptual disturbances (score 0–3); hallucinations (score 0–3); delusions (score 0–3); psychomotor behavior (score 0–3); cognitive status (score 0–4); physical disorder (score 0–2); sleep-wake cycle disturbance (score 0–4); liability of mood (score 0–3); and variability of symptoms (score 0–4). The sum of all item scores comprised the total DRS; the maximum possible score was 32 points. A cut-off of >12 has been suggested to distinguish patients with delirium from patients with other neuropsychiatric disorders [24]. The DRS was performed once daily before midday, independent of additional analgesia or sedation. Delirious behavior was recorded every shift by the bedside nurse. The DRS was performed until there were two consecutive assessments with a DRS score ≤12. Postoperative delirium was diagnosed using the Diagnostic and Statistical Manual of Mental Disorders, 4^th^ ed. [25] criteria and all available data, including from the DRS and chart, by a single psychiatrist.

### Anesthetic Management and Evaluation of Arteriosclerosis in the Aorta

Diazepam and fentanyl were used to induce and maintain anesthesia. They were supplemented as needed with isoflurane or sevoflurane during surgery. Surgical access to the heart was made through a standard median sternotomy in all patients. Surgery with CPB was performed using a membrane oxygenator and roller pump under alpha-stat pH management and moderate hypothermia (32 to 34°C), as described previously [26]. Blood from cardiotomy suction catheters was separated from the pump circuit and washed with a cell saving device. The mean arterial pressure was maintained between 50 and 70 mmHg and the difference between rectal and CPB perfusate temperature was restricted to 5 to 6°C during rewarming.

All patients underwent epiaortic ultrasound scans at the time of surgery to screen for atherosclerosis of the ascending aorta. We divided the ascending aorta from the aortic valve to the innominate artery into three segments and assessed intimal thickening off-line on videotape records, as reported previously [27]. The degree of atherosclerosis in the ascending aorta was graded as: normal or mild (<3 mm), moderate (atheroma ≥3 mm in one segment of the ascending aorta), or severe (atheroma ≥3 mm in 2 or all 3 segments, or mobile atheroma). All patients were assessed by a single echocardiograph technician. Decisions to modify cannulation, clamping, proximal graft anastomotic sites and/or cardioplegia cannulation sites were determined by a single cardiac surgeon based on findings from real-time imaging.

### Statistical analysis

Differences in continuous variables between groups with and without POCD were tested for significance using one-way analysis of variance and Student's t-test. Significance among categorical variables was determined using Fisher's exact test. A P value <0.05 was considered significant. Stepwise logistic regression analysis was performed to assess for predictors of POCD. Variables included in the multivariate model included all those found to be associated with this condition at <0.20 on univariate analysis. Odds ratios (OR) and 95% confidence interval (CI) were calculated for each variable. Goodness of fit was assessed by the Hosmer-Lemeshow statistic. Tests were performed using the SAS Institute, Inc., statistics package (version 9.1; Cary, NC, USA).

## Results

The 28 patients enrolled in the study had average age of 73±8 years. The study group included 9 women and 19 men. Postoperative cognitive dysfunction was identified in 8 of the 28 patients (28.6%). The incidence of postoperative delirium was 10.7% (3 of 28 patients). There was no statistically significant differences in POCD for delirium incidence (1 patient, 12.5% with POCD vs. 2 patients, 10% without POCD, P = 0.847). Delirium was observed within 48 h after surgery among those who had delirium. Demographic and operative data for these patients are listed in Table 1, based on the presence or absence of POCD. Patients with POCD had significantly more education than patients without POCD.

**Table 1:** Characteristics of Patients with and without Postoperative Cognitive Dysfunction.

	POCD n = 8 (29%)	No POCD n = 20	*p* Value
Age (years)	75.9±8.1	71.4±7.2	0.159
≥75	5 (63)	5 (25)	0.136
65≤74	2 (25)	13 (65)	
Gender (male/female)	6/2	13/7	1.000
Education (years)	12.6±3.0	10.3±2.2	0.028
Hypertension	5 (63)	15 (75)	0.651
Diabetes mellitus	3 (37)	6 (30)	1.000
Peripheral arterial disease	1 (13)	1 (5)	0.497
History of atrial fibrillation	4 (50)	5 (25)	0.372
Ejection fraction	59.6±12.6	65.8±14.2	0.297
Preoperative medications			
Aspirin	5 (63)	13 (65)	1.000
Statins	4 (50)	9 (45)	1.000
β-blockers	4 (50)	6 (30)	0.400
Surgical procedure			
CABG	1 (13)	8 (40)	0.061
CABG/Valvular	3 (37)	1 (5)	
Valvular	4 (50)	11 (55)	
CPB time (min)	202±57	183±74	0.529
Cross-clamp time (min)	116±34	119±41	0.861

Continuous variables are presented as mean ± SD and categorical variables are presented as frequency (percentage). CABG  =  coronary artery grafting; CPB  =  cardiopulmonary bypass.

Raw neuropsychological test scores are reflected in Table 2 for both baseline and 2 weeks after surgery. Baseline neuropsychological test scores were similar between the 2 groups. When neuropsychological tests were examined individually, patients with POCD had significantly lower performances on the MMSE (global cognitive function), Digit Symbol test, Trail Making test A (complex attention), Kana Pick-out test, and Trail Making test B (executive function).

**Table 2:** Neuropsychological Results for Patients with and without Postoperative Cognitive Dysfunction.

Cognitive domain	Instrument	POCD n = 8	No POCD n = 20	*p* Value
Composite measure	**MMSE**			
	preoperative	26.8±1.9	26.9±1.9	0.902
	follow-up	24.4±2.1	26.8±3.0	0.050
	Δ^a^	−2.4±1.7	−0.1 ±2.4	0.024
Verbal memory	**Digit Span Forward**			
	preoperative	8.1±1.1	7.1±1.7	0.132
	follow-up	8.1±1.7	6.9±1.9	0.109
	Δ^a^	0.0 ±1.3	−0.3 ±1.1	0.604
	**Digit Span backward**			
	preoperative	5.5±1.3	4.6±1.4	0.121
	follow-up	5.0±1.6	4.4±1.4	0.302
	Δ^a^	−0.5±1.3	−0.3±1.2	0.624
Complex attention	**Digit Symbol Test**			
	preoperative	32.8±8.3	32.6±12.9	0.976
	follow-up	22.6±8.1	34.0±12.0	0.022
	Δ^a^	−10.1±4.8	1.4±4.4	0.001
	**Trail Making Test A time (sec)**			
	preoperative	68.8±32.8	59.7±28.5	0.473
	follow-up	94.1±49.9	62.2±25.0	0.031
	Δ^a^	25.4±38.9	2.5 ±13.4	0.026
Executive function	**Kana Pick-out Test**			
	preoperative	22.6±8.2	23.8±10.7	0.782
	follow-up	14.4±8.0	27.7±12.6	0.011
	Δ^a^	−8.3±4.3	3.9±5.4	0.001
	**Trail Making Test B time (sec)**			
	preoperative	189±75	173±102	0.690
	follow-up	298±170	179±89	0.023
	Δ^a^	109±102	6.6±42	0.001

MMSE  =  Mini-Mental State Examination.

a Change between preoperative and follow-up test scores.

Preoperative MRI findings of the brain and ascending aortic atherosclerosis are shown in Table 3. Patients with POCD had significantly higher rates of white matter lesions on MRI, and more loss of gray matter in the MTL than patients without POCD.

**Table 3:** Prevalence of Gray Matter Loss in the Medial Temporal Lobe, Intracranial and Carotid Artery Stenosis, and Atherosclerosis of the Ascending Aorta.

	POCD n = 8	No POCD n = 20	*p* Value
**Gray matter loss of MTL (Z score)**	2.0±0.9	1.1±0.9	0.027
Hardly any gray matter loss	1 (12)	13 (65)	0.044
Some gray matter loss	3 (38)	4 (20)	
Considerable gray matter loss	3 (38)	1 (5)	
Prominent gray matter loss	1 (12)	2 (10)	
**MRI (White matter lesion)**			
Absence	3 (38)	9 (45)	0.028
Punctate foci	0	6 (30)	
Beginning confluence of foci	1 (12)	4 (20)	
Large confluent areas	4 (50)	1 (5)	
**MRI (brain)**			
No infarcts	4 (50)	17 (85)	0.149
Single infarct	1 (12)	1 (5)	
Multiple infarcts, single location	3 (38)	2 (10)	
Multiple infarcts, multiple location	0	0	
**MRA (carotid arteries)**			
Normal or mild	8 (100)	19 (95)	1.000
Moderate	0	0	
Severe	0	1 (5)	
**MRA (cerebral arteries)**			
Normal or mild	8 (100)	19 (95)	1.000
Moderate	0	1 (5)	
Severe	0	0	
**Aortic atherosclerosis**			
Normal or mild	5 (63)	17 (85)	0.424
Moderate	1 (12)	1 (5)	
Severe	2 (25)	2 (10)	

MTL  =  medial temporal lobe; MRI  =  magnetic resonance imaging; MRA  =  magnetic resonance angiography.

Stepwise logistic regression identified the presence of gray matter loss in the MTL (OR per grade 2.90; 95% CI, 1.07–7.82; P = 0.036) and cerebral infarctions detected by MRI (per grade 3.42; 95% CI, 1.01–11.7; P = 0.049) as being associated with POCD after cardiac surgery. The final study model had an area under the receiver operating characteristic curve (“c” index) of 0.822 with an adjusted Hosmer-Lemeshow test statistics of 0.410.

## Discussion

This study demonstrated that patients who experienced POCD within 2 weeks after cardiac surgery had significantly greater loss of gray matter in the MTL than patients who did not experience POCD. The findings suggested that patients with POCD had significantly higher rates of white matter lesions on MRI, and had attained more education than patients without POCD. Multivariate analysis revealed that gray matter loss in the MTL and cerebral infarctions were independent predictors of POCD.

Several structural MRI studies have demonstrated that loss of gray matter in the MTL, including the hippocampus and entorhinal cortex, has been observed frequently in patients with AD and those with MCI [10], [11]. VSRAD is a VBM software program with Z score map in the bilateral MTL and it discriminates AD at an early stage of amnestic-type MCI and healthy controls [15]. Our VSRAD estimation revealed that cardiac patients who experienced POCD potentially lose gray matter in the MTL (average Z score, 2.0). Recent studies have found MCI patients vulnerability to postoperative cognitive impairment, both in the higher relative risk of a POCD-like change and the significant change in the composite cognitive parameter during the first evaluation interval [9], [28]. Thus, our data support the notion that POCD contributes to understanding the etiology of POCD and analysis of risk factors leading to progression of dementia.

Results from our data showed that cerebral infarctions made independent and additive contributions to POCD. Goto et al. [26] found that patients undergoing cardiac surgery have a high prevalence of cerebral small vessel disease and that such patients have worse postoperative cognitive outcomes than patients with normal preoperative findings. Cerebral small vessel disease is the most common pathology underlying vascular dementia, and is a major cause of lesser degrees of vascular cognitive impairment [29]. Additionally, several studies have suggested MTL are smaller in patients with cerebral small vessel disease than in cognitively normal subjects [30], [31]. These reports suggested that the pathogenesis of gray matter loss of MTL may reflect a combination of neuronal degeneration and ischemic pathologies. Therefore, estimating the degree of gray matter loss in the MTL might have reflected vulnerability to brain ischemia in patients who have cardiac surgery.

Recently, several studies have suggested that a baseline cognitive diagnosis may identify additional patients at risk for POCD [4]–[8]. Clearly, the baseline level of cognition is important because measuring additional decline may be more difficult if cognition already is impaired before surgery. Silverstein et al. [5] used the International Study of Postoperative Cognitive Dysfunction (ISPOCD) database to explore patterns of deterioration in patients with preoperative cognitive impairment. The highest degree of deterioration at 1 week after surgery was seen in tests thought to assess attention and cognitive speed. In patients with preoperative cognitive impairment, deterioration in memory function was significantly less common. Bekker et al. [28] in their retrospective study also found that surgery negatively affects the domain of attention and concentration in patients with a preoperative diagnosis of MCI. In the present study, patients with POCD scored significantly lower on the attention and executive function. Considering the limitations of these studies, our findings are remarkably similar. However, impaired memory and learning, and atrophy in the hippocampus, are highly consistent because memory and learning depend heavily on the hippocampus and its integrity [32]. This discrepancy in impairment profiles could indicate that the pathophysiology of brain damage in patients after cardiac surgery differs from that of patients with AD and those with MCI. Understanding these discrepancies is an important challenge for future studies.

It was a surprise that our patients with POCD had attained significantly more education than patients without POCD. Preoperative cognitive performance was similar between patients with and without POCD. Education has been proposed as a protective factor that can reduce the risk of developing dementia [33]. It is also well known that a lower level of education is one of the consistent predictors of POCD [34], [35]. One plausible explanation for our findings might be that higher education can appear to mask the clinical expression of a higher degree of neurodegeneration [36]. However, such reserve capacity is difficult to measure, and comparable education by no means guarantees comparable levels of reserve.

There were several limitations to the present analysis. First, the small sample size may limit wide applicability. Second, although no definitive standard exists, defining POCD by establishing a threshold value on cognitive test scores is arbitrary. Our use of the neurocognitive domains is less conservative than some other study groups have applied. Therefore, we analyzed raw neuropsychological test scores as well. Third, postoperative assessments took place at 2 weeks. It might be concluded that the decline in function after surgery was only transient and reversible in the majority of patients. However, it has been reported that cognitive decline at discharge is a predictor of long-term cognitive dysfunction [18]. A reevaluation of patients after one year would help to address this issue. Fourth, no MRI were performed after surgery. Therefore, the relationship between POCD and postoperative new cerebral ischemic lesions on MRI is uncertain [37]. The final limitation of the study was our inability to assess postoperative risk factors, such medication side effects, mechanical ventilation and pain. Although there are routine postoperative pain and sedation protocols in our intensive care unit, it is difficult to distinguish these medications as a cause or effect of POCD; and this study was not designed to answer that question.

In conclusion, this preliminary study found that reduced gray matter in the bilateral MTL and white matter lesions existed in brains of elderly cardiac surgery patients who experienced POCD. These abnormalities in brain structure may be related to the expression of a POCD. Thus, our results emphasize the need for a more comprehensive examination of surgery, cognition, and structural changes in the brain.
